# Assessment of CD27 expression on T-cells as a diagnostic and therapeutic tool for patients with smear-negative pulmonary tuberculosis

**DOI:** 10.1186/s12865-021-00430-y

**Published:** 2021-06-27

**Authors:** Feifan Xu, Haiyun Zhang, Xiaoyan Si, Junlin Chen, Yuhao Chen, Xiaopeng Cui, Yongwei Qin

**Affiliations:** 1 Department of Pathogen Biology, School of Medicine, Nantong University, Nantong, 226001 China; 2 Department of Clinical Laboratory, The Sixth People’s Hospital of Nantong, Nantong, 226011 China; 3 Department of Tuberculosis, The Sixth People’s Hospital of Nantong, Nantong, 226011 China; 4 Department of General Surgery, Affiliated Hospital of Nantong University, Nantong, China

**Keywords:** Smear-negative pulmonary tuberculosis, CD27, Diagnosis, Therapy

## Abstract

**Background:**

There is a global focus on illness diagnosis in smear-negative and latent tuberculosis infectious populations (SN-TB and LTBI). CD27 has been suggested to play a direct role in active TB. Little is known about smear-negative individuals. Here, we tried to investigate whether it has a role in smear-negative populations. The expression of CD27 and MTB-specific CD27 in CD4^+^ T cells (“CD27^−^CD4^+^” and “CD27^−^IFN-γ^+^CD4^+^”) was evaluated in MTB-unexposed controls (HC), TB contacts (TB-C) and SN-TB individuals by flow cytometry. The sensitivity, specificity and AUC (area under curve) of “CD27^−^IFN-γ^+^CD4^+^” cells to distinguish SN-TBs from HCs and TB-Cs were determined by receiver operating characteristic (ROC) curve analysis. The clinical index was selected from the clinical laboratory and evaluated for correlation with “CD27^−^IFN-γ^+^CD4^+^” cells by Spearman statistical analysis.

**Results:**

We observed that the percentages of “CD27^−^IFN-γ^+^CD4^+^” cells were significantly increased in the SN-TB group compared with the HC and TB-C groups (AUC was 0.88, sensitivity was 82.14%, specificity was 80.00%, and *P* < 0.0001). The percentage of “CD27^−^IFN-γ^+^CD4^+^” cells was negatively correlated with WBC (white blood cell count) (r = − 0.3019, *P* = 0.0182) and positively correlated with IgE (immunoglobulin E) (r = 0.2805, *P* = 0.0362). Furthermore, “CD27^−^IFN-γ^+^CD4^+^” cells were significantly decreased, especially in the > 50 years group, after clinical treatment.

**Conclusion:**

The present results demonstrated that the percentage of “CD27^−^IFN-γ^+^CD4^+^” cells might be a conceivable molecular indicator in the diagnosis of SN-TB and was influenced by its outcome of therapy.

**Supplementary Information:**

The online version contains supplementary material available at 10.1186/s12865-021-00430-y.

## Background

*Mycobacterium tuberculosis* (MTB) and its related disease tuberculosis (TB) are infectious diseases with a global focus [1]. Although both vaccines and anti-TB drugs are popular, TB has re-emerged unexpectedly [2]. Globally, almost 20–50% of TB cases are smear-negative TB (SN-TB) without identifiable bacteriological evidence and are difficult to clinically diagnose [3–5]. According to previous studies, chest X-ray (CXR) and culture were suggested for SN-TB diagnosis, but the pooled sensitivity and specificity were only 61 and 69%, respectively [6]. Some diagnostic tests, including microscopic observation drug susceptibility assay (MODS) and Xpert analysis, were also performed in the clinic, but all of them have limitations [7]. Recently, immunological assays based on T cell-mediated IFN-gamma responses (i.e., QuantiFERON-TB Gold and T-SPOT. TB) have been proven to be useful in TB diagnosis with a limitation in distinguishing between latent and active TB [8, 9]. Hence, a new examination for SN-TB is urgently needed.

To a great extent, the cell-mediated immune response is involved in and regulates MTB, and CD4 T cells are thought to play a momentous role in controlling MTB infection [10]. CD27, a member of the TNF-receptor superfamily, is inextricably expressed on the mature pathogen-specific CD4^+^ T cell surface [11]. Previous studies have verified CD27 as an immune biomarker in active TB and pulmonary destruction. Decreased CD27 facilitated differentiated effector T cell formation and generated many cytokines under inflammatory and/or antigenic stimulation. In infectious diseases, “CD27^−^CD4^+^” cells accumulated more in peripheral blood and infectious sites, interpreted as a homing process for designated cell subtypes migrating according to infectious organization [12]. Recently, a new strategy based on the CD27 molecular marker was used for active TB diagnosis and could differentiate active TB and latent TB infection (LTBI) by examining MTB-specific CD27 expression in CD4^+^ T cells [13]. There is a similar case for detecting the median fluorescence intensity (MFI) ratio for CD27 expression after or without PPD or MTB-specific antigen (ESAT-6/CFP-10) stimuli [14]. These findings provide a possibility for new research on the treatment strategy and underlying mechanism of TB.

In this study, we focused on evaluating several T cell subcohorts presenting different TB conditions based on smear-negative individuals (aetiology examination negative, T-SPOT positive), especially in special populations such as doctors, nurses and clinical laboratory personnel in TB-specific hospitals. Our studies provided a rapid diagnosis for this population by detecting MTB-specific CD27 expression and secretion of IFN-γ in CD4^+^ T cells. We found that “CD27^−^IFN-γ^+^CD4^+^” T cells accumulated in SN-TB peripheral blood compared with that of HC individuals and TB-Cs with a correlation with WBC and IgE. Furthermore, we also observed that “CD27^−^IFN-γ^+^CD4^+^” cell expression was correlated with the effect of anti-TB treatment.

## Results

### Characteristics of the study population

Patient characteristics are shown in Table 1. In the present study, the sex distribution was approximately equal, the SN-TB (45.14 ± 13.52 years) and HC (44.24 ± 15.15 years) groups were older and had a lower BMI (20.26 ± 2.31 kg/m^2^, 20.89 ± 2.28 kg/m^2^) than the TB-C (38.34 ± 9.42 years, 21.54 ± 2.43 kg/m^2^) group. In the study, SN-TB patients exhibited decreased WBC, RBC and PLT counts (5.34 ± 2.12 × 10^9^/L, 3.73 ± 0.84 × 10^12^/L, 240.89 ± 85.52 × 10^9^/L) compared to the TB-Cs (6.87 ± 2.15 × 10^9^/L, 4.37 ± 0.52 × 10^12^/L, 334.54 ± 55.67 × 10^9^/L) and HCs (6.62 ± 2.08 × 10^9^/L, 4.31 ± 0.60 × 10^12^/L, 307.24 ± 89.91 × 10^9^/L). In addition, there was no difference in HGB, IgA, IgG, IgM, IgE, ESR or CRP among the three groups.

**Table 1:** Characterization of groups included in the study

Characteristic	HC	TB-C	SN-TB	*P* value
	*n* = 34	n = 46	*n* = 56	
Sex (male/female)	20/14	27/19	27/29	0.4844
Age (years)	44.24 ± 15.15	38.34 ± 9.42	45.14 ± 13.52	0.0215*
BMI (kg/m2)	20.89 ± 2.28	21.54 ± 2.43	20.26 ± 2.31	0.0268*
TSPOT.TB (+)	0 (0%)	46 (100%)	56 (100%)	< 0.0001***
With BCG injected	33 (97.06%)	46 (100%)	52 (92.86%)	0.1591
WBC (×109/L)	6.62 ± 2.08	6.87 ± 2.15	5.34 ± 2.12	0.0008**
RBC (×1012/L)	4.31 ± 0.60	4.37 ± 0.52	3.73 ± 0.84	< 0.0001***
HGB (g/L)	128.35 ± 16.96	129.39 ± 11.63	121.55 ± 23.37	0.0738
PLT (×109/L)	307.24 ± 89.91	334.54 ± 55.67	240.89 ± 85.52	< 0.0001***
IgA (g/L)	2.41 ± 0.98	2.78 ± 0.98	2.56 ± 1.43	0.3754
IgG (g/L)	11.95 ± 3.11	10.91 ± 3.00	10.26 ± 4.97	0.1508
IgM (g/L)	2.33 ± 0.89	2.12 ± 1.17	2.16 ± 1.95	0.8188
IgE (IU/mL)	35.53 ± 88.72	59.42 ± 165.22	84.05 ± 176.32	0.3491
ESR (mm/h)	32.35 ± 47.66	27.50 ± 42.56	41.75 ± 35.03	0.2079
CRP (mg/L)	21.92 ± 40.34	21.88 ± 38.90	31.44 ± 43.61	0.4171

Significant value: *P < 0.05; **P < 0.01; *** *P* < 0.00001

### Smear-negative TB patients have higher percentages of “CD27^−^IFN-γ^+^CD4^+^” cells than do MTB-unexposed individuals and TB contacts

**Fig. 1:**
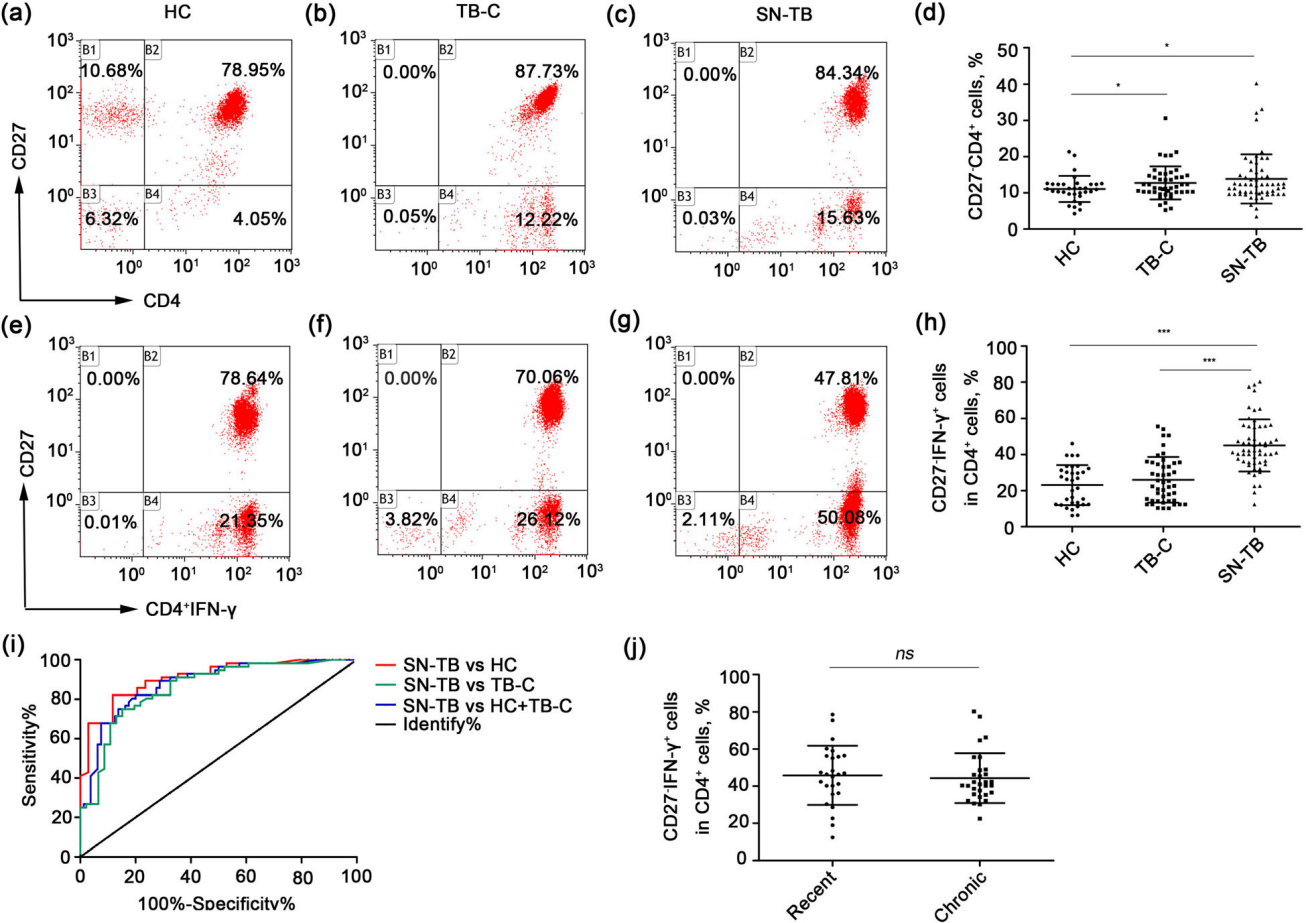
Smear-negative TB patients have higher percentage of CD27^−^IFN-γ^+^ cells in their peripheral blood. **a-d** The percentage of CD27 in CD4 T cells population. **e-h** The percentage of “CD27^−^IFN-γ^+^CD4^+^” cells population by a flow cytometry. **i** ROC-curve of “CD27^−^IFN-γ^+^CD4^+^” percentages for discriminating SN-TB patients from HC or (and) TB-C. **j** The percentage of “CD27^−^IFN-γ^+^CD4^+^” cells population in recent and chronic SN-TB patients. ^***^P < 0.0001

To research the role of CD27 in SN-TB patients, the percentages of CD27^−^ cells within the total population of CD4^+^ T cells (“CD27^−^CD4^+^” cells) were analysed by a flow cytometer. As shown in Fig. 1a-d, compared with HC and TB-C subjects, SN-TB patients had a high percentage of “CD27^−^CD4^+^” T cells. There was no significant difference between the TB-C and SN-TB patients. To further identify the CD27^−^ expression of MTB-specific CD4^+^ T cells, “CD27^−^IFN-γ^+^CD4^+^” cells were evaluated. As shown in Fig. 1e-h, the percentages of “CD27^−^IFN-γ^+^CD4^+^” cells were significantly higher in SN-TB patients but were similar between HC individuals and TB-C. A ROC curve (Fig. 1i) showed that the expression of “CD27^−^IFN-γ^+^CD4^+^” could distinguish the SN-TB patients from the HC and TB-C individuals, and the AUC was 0.88. Moreover, the sensitivity was 82.14%, and the specificity was 80.00%, with a 95% CI of 0.8214 to 0.9371 (shown in Table 2). In addition, there was also no significant difference between recent and chronic infection in SN-TB patients (*P* > 0.05, Fig. 1j). Thus, the expression of CD27^−^IFN-γ^+^ might be a conceivable molecular indicator for detecting SN-TB patients.

**Table 2:** Distinction smear-negative TB from all health participants by detecting “CD27^−^IFN-γ^+^CD4^+^” cells expression

	Sensitivity%	Specificity%	AUC	95% CI	*P*
SN TB vs HC and TB C	82.14	88.24	0.88	0.8214 to 0.9371	< 0.0001***
SN TB vs HC	82.14	80.00	0.91	0.8491 to 0.9681	< 0.0001***
SN TB vs TB C	80.36	76.09	0.86	0.7839 to 0.9312	< 0.0001***

***Significant value (*P* < 0.0001)

### In smear-negative TB patients, the expression of “CD27^−^IFN-γ^+^CD4^+^” cells in peripheral blood is correlated with WBC counts and IgE expression

**Fig. 2:**
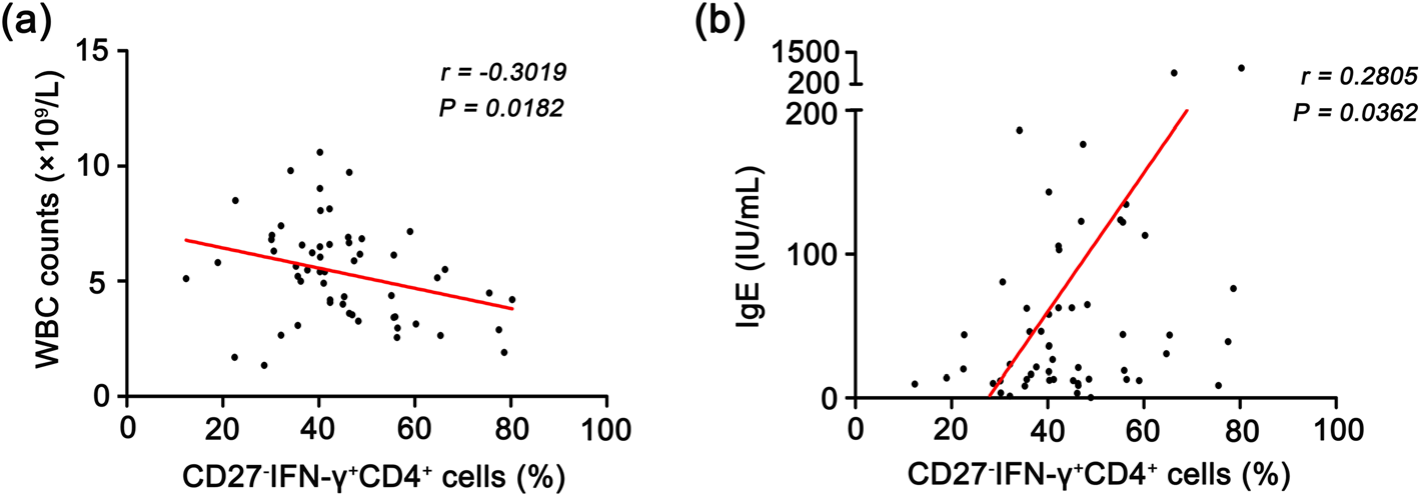
Relationship between the percentage of “CD27^−^IFN-γ^+^CD4^+^” expression and clinical laboratory index. **a** Relationship between the percentage of “CD27^−^IFN-γ^+^CD4^+^” expression and WBC counts. **b** Relationship between the percentage of “CD27^−^IFN-γ^+^CD4^+^” expression and IgE

To evaluate whether the expression of “CD27^−^IFN-γ^+^CD4^+^” correlated with the patients’ immune state, we analysed “CD27^−^IFN-γ^+^CD4^+^” expression with clinical laboratory parameters. As shown in Fig. 2a and b, the percentages of “CD27^−^IFN-γ^+^CD4^+^” were negatively correlated with WBC counts (*r* = − 0.3019, *P* = 0.0182) and positively correlated with IgE (*r* = 0.2805, *P* = 0.0362). There was no significant correlation of “CD27^−^IFN-γ^+^CD4^+^” cells with the inflammation index (ESR and CRP) or other immunoglobulins (IgG, IgA and IgM) (Supplemental Fig. 2, *P* > 0.05).

### In smear-negative TB patients, the distribution of “CD27^−^IFN-γ^+^CD4^+^” cells in peripheral blood is associated with the effect of the therapy

**Fig. 3:**
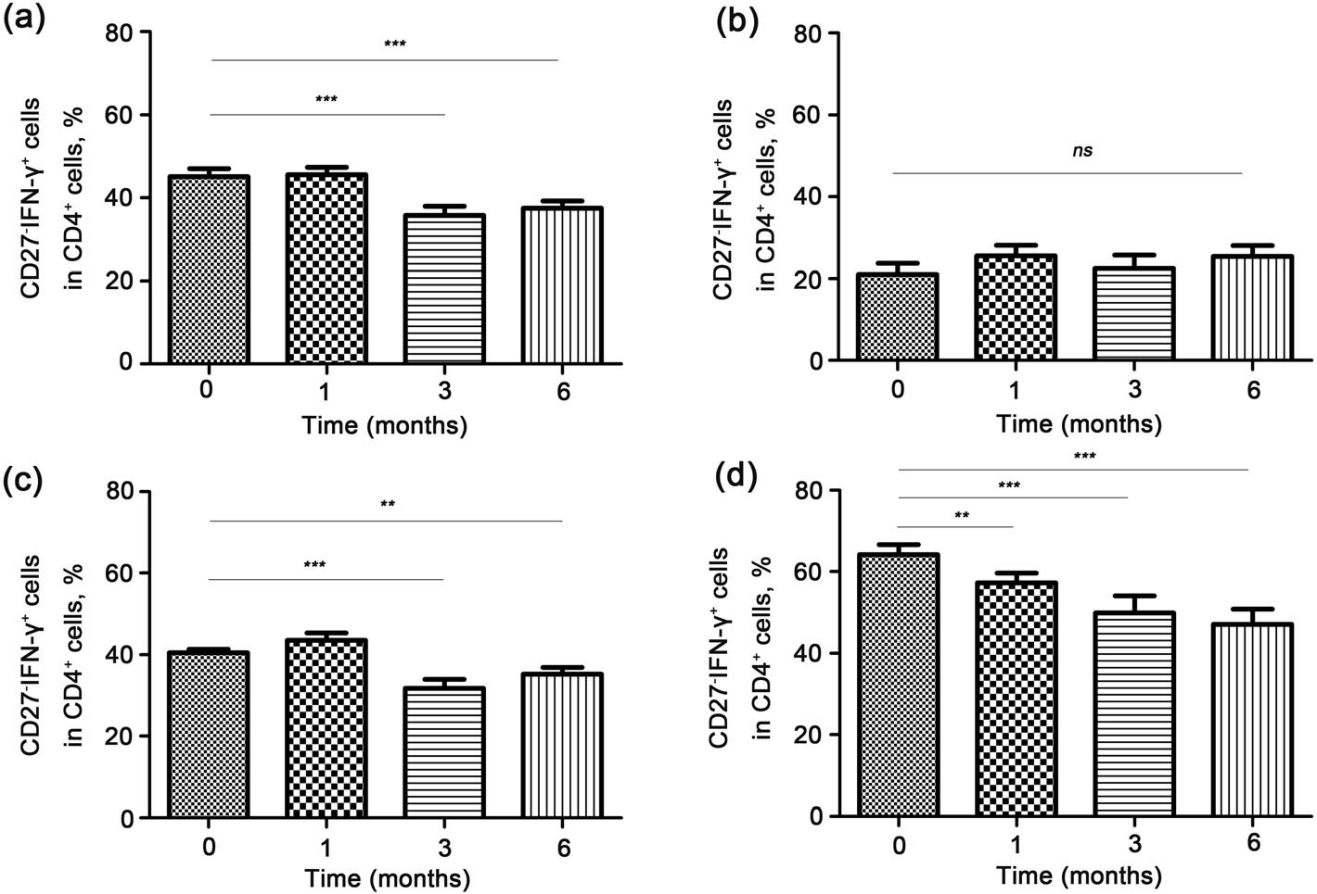
Distribution and outcome of “CD27^−^IFN-γ^+^CD4^+^” in peripheral blood after normal anti-TB therapy. **a** Total percentage of “CD27^−^IFN-γ^+^CD4^+^” cells after treatment. **b** The percentage of “CD27^−^IFN-γ^+^CD4^+^” in < 30 years group. **c** The percentage of “CD27^−^IFN-γ^+^CD4^+^” in 30–50 years group. **d** The percentage of “CD27^−^IFN-γ^+^CD4^+^” in > 50 years group. Data are presented as the mean ± standard deviation of at least three independent experiments. ^*^P < 0.05 vs. no therapy at 0 month. **P < 0.01 vs. no therapy at 0 month. ^***^P < 0.0001 vs. no therapy at 0 month

To evaluate the expression of “CD27^−^IFN-γ^+^CD4^+^” cells in anti-TB therapy, 56 SN-TB patients enrolled in this study received normal anti-TB therapy. The effect of the treatment was evaluated by clinician and radiologist. In the therapeutic process, blood was collected from all patients into EDTA-anticoagulation tubes. Figure 3a suggests that the percentage of “CD27^−^IFN-γ^+^CD4^+^” cells was significantly decreased at 3 and 6 months (*P* < 0.05, compared to 0 months). To further investigate the changes in “CD27^−^IFN-γ^+^CD4^+^” in different age groups, we divided all the patients into three groups (< 30 with 5 cases, 30–50 with 36 cases and > 50 groups with 15 cases). As shown in Fig. 3b, there were no obvious differences in the < 30 group (*P* > 0.05, compared to 0 M) which might be influenced by the limited numbers of cases. Significant differences occurred in the 3 M and 6 M groups at 30–50 M (Fig. 3c, *P* < 0.05, compared to 0 M) and all stages in the > 50 group (Fig. 3d, *P* < 0.05, compared to 0 M). These results indicated that age might be a key factor in the treatment of SN-TB patients.

## Discussion

In this study, we demonstrated that the percentages of “CD27^−^IFN-γ^+^CD4^+^” T cells rather than “CD27^−^CD4^+^” cells in SN-TB patients were higher than those of HC and TB-C individuals. The ROC curve indicated that the AUC was 0.88 (the sensitivity was 82.14%, and the specificity was 80.00%, with a 95% CI of 0.8214 to 0.9371). Moreover, the expression of “CD27^−^IFN-γ^+^CD4^+^” correlated with WBC counts and IgE expression. In addition, we also evaluated the changes in CD27^−^IFN-γ^+^ in the whole CD4^+^ T-cell population after normal anti-TB treatment and found that it was significantly decreased in the 30–50-year-old group and > 50-year-old group, particularly at 3 months and 6 months.

Protection against MTB infection is largely dependent on T cell responses. Although γδ T and CD8^+^ cells play important roles in protection, CD4^+^ T cells capable of producing and activating macrophage antimycobacterial functions are thought to be the major immune regulator of MTB [15–17]. Recently, researchers focused their attention on techniques that could more accurately characterize and measure the CD4^+^ T cell response, such as flow cytometry [18, 19]. In this study, we mainly used flow cytometry to analyse the various molecular characteristics of PBMCs (peripheral blood mononuclear cells). Our data indicating no significant difference in TB contacts and smear-negative TB patients suggested that the expression of CD27 in CD4^+^ T cells could not differentiate healthy, latent TB infection and smear-negative TB.

Previous studies have verified some cytokine secretion profiles from CD4^+^ T cells in patients with tuberculosis, such as IFN-γ^+^, TNF-α, IL-2 and CD27 [18, 20, 21]. CD27 is a member of the TNF-receptor superfamily participating in the genesis and development of TB [19, 22, 23]. Nikitina et al. suggested that CD27^−^IFN-γ^+^ cells play an important role in assessing TB activity, lung destruction, and tissue repair after TB therapy in the majority of smear-positive TB patients [12]. Schuetz et al. indicated that HIV+ patients have higher expression of CD27^−^IFN-γ^+^CD4^+^ T cells than HIV- persons, suggesting that accumulation of MTB-specific CD27^−^CD4^+^ cells might reflect the degree of MTB replication, thus affecting subclinical *M. tuberculosis* infection. The loss of CD27 expression on IFN-γ^+^CD4^+^ T cells in active TB disease in one HIV-infected patient lends further support to this hypothesis [24]. In addition, the TAM-TB assay, which evaluates the ratio of the median fluorescence intensity of CD27 in the whole CD4^+^ T-cell population to that of CD27 in the MTB-specific IFN-γ^+^ CD4^+^ T cells, has a vital role in distinguishing between tuberculosis in LTBI in adults and children [25]. Nevertheless, studies on SN-TB are rare. Our study focused on 56 smear-negative TB patients and found that CD27^−^IFN-γ^+^CD4^+^ was higher in SN-TB patients than in MTB-unexposed individuals and TB contacts. The following studies indicated that “CD27^−^IFN-γ^+^CD4^+^” correlated with the treatment of TB. Examinations based on T cell responses (TST, T-SPOT, IGRAs) have been widely researched and used in the auxiliary diagnosis of TB [25–27]. Recently, studies have indicated that they have no roles in the discrimination between latent infection and active TB [28]. Our study verified that CD27^−^IFN-γ^+^ in the whole CD4^+^ T-cell population could not distinguish TB-infected patients from healthy controls.

## Conclusions

We demonstrated the increased expression of “CD27^−^IFN-γ^+^CD4^+^” cells in smear-negative TB patients and evaluated its changes after normal anti-TB therapy. These data suggested that “CD27^−^IFN-γ^+^CD4^+^” T cells could be used to assist the diagnosis of TB and more accurately evaluate the treatment outcome of smear-negative TB.

## Methods

### Study subjects and ethics statement

All data were collected in our hospital from July 2016 to August 2018, and the study was approved by the hospital ethics committee. All participants enrolled in the study provided written informed consent and did not have any other infectious diseases, such as hepatitis or HIV-1 infection.

### Criteria for inclusion and exclusion

TB was diagnosed based on clinical and radiographic evidence. The HC group included 34 participants with no exposure to MTB and no clinical characteristics of TB, and the PPD test was negative (Table 1). TB-C (*n* = 46, Table 1) was the group of doctors and nurses working closely with TB patients for at least 1 year. Of the fifty-six SN-TB patients, all had negative detection of sputum at least 3 times and sputum negative-culture results (Table 1). The patient flow diagram is shown in Supplemental Fig. 1. All fifty-six smear-negative TB patients included in our study had peripheral blood collected and sputum collected at least three times for Ziehl-Neelsen acid-fast stain (Zhuhai DL Biotech Co., Ltd., Guangdong, China). Sputum culture was carried out by BACTEC MGIT 960 (Becton Dickinson, Sparks, USA) liquid culture isolates using the GenoTypeH test system (Hain Lifescience, Nehren, Germany) and improved L-J culture medium (Baso Biotech Co., Ltd., Guangdong, China). The applied SN-TB diagnostic criteria followed the WHO guidelines based on a combination of clinical symptoms, chest radiological evidence, histological observations, other pathogenic detection of sputum and BALF specimens from bronchoscopy and a decision by the attending clinician that the patient had a satisfactory response to all courses of anti-TB therapy [29]. To identify the role of “CD27^−^IFN-γ^+^CD4^+^” T cells, peripheral blood was collected from all SN-TB outpatients, and CD27^−^IFN-γ^+^ expression was detected. To compare CD27 expression after TB therapy, all smear-negative patients enrolled in this study received normal anti-TB treatments. Anti-tuberculosis therapy consisted of isoniazid, rifampin, pyrazinamide, and ethambutol daily for 2 months (M) and then isoniazid and rifampin daily for 4 M. Peripheral blood was collected at 0 M, 1 M, 3 M and 6 M. Participants excluded when they were TB aetiology positive, death, combined with other serious infectious disease or lose of follow-up in the research course.

### Data extraction

T-SPOT (tuberculosis infectious T-lymphocyte spot assay, Oxford Immunotec, Abingdon, UK) results were obtained from the clinical laboratory. WBC (white blood cell count), RBC (red blood cell count), HGB (haemoglobin), and PLT (platelet count) assays were carried out using a Mindray BC-6900 hematology analyser (Shenzhen Mindray Bio-Medical Electronics Co., Ltd., China). IgA (immunoglobulin A), IgG (immunoglobulin G), IgM (immunoglobulin M), IgE (immunoglobulin E), and CRP (C-reactive protein) were obtained from Beckman Coulter 5800 series clinical chemistry analyzer (Tokyo, Japan). ESR (erythrocyte sedimentation rate) were acquired from the Clinical Laboratory Department of our hospital measured by Eriline AR Linear (Barcelona, Spain).

### Flow cytometry analysis

For detection of the whole blood cell surface markers “CD27^−^CD4^+^” T cells, 100 μl of freshly isolated blood anticoagulated by ethylenediaminetetraacetic acid (EDTA) was stained by FITC-anti-CD27 mAbs and PE-anti-CD4 (BD Biosciences, San Jose, USA) at room temperature for 10 min. Red blood cells were lysed by red cell lysis solution (BD Biosciences, San Jose, USA). Cells were collected, washed and analysed by a Beckman Coulter FC500 flow cytometry analysis system (USA). For “CD27^−^IFN-γ^+^CD4^+^” cells, 0.5 ml of freshly isolated whole blood was diluted with 0.5 ml of RPMI medium and incubated with the specific MTB mixed antigen (浓度Biaoyuan Biotech Co. Ltd., Jiangsu, China) at 37 °C in a 5% CO_2_ incubator. Whole blood diluted without antigen stimulation was used as the negative control. After 4 h of culture, GolgiPlug (BD Biosciences) was added and cocultured overnight. Cells were stained with PE-anti-CD4 and FITC-anti-CD27 mAbs for 10 min, and red blood cells were lysed by red cell lysis solution. After centrifugation for 15 min at 1500×g, the supernatant was discarded, and cells were treated with BD FACS Lysis solution and BD FACS Permeabilizing solution II, stained with APC-anti- IFN-γ mAbs (BD Biosciences, San Jose, USA) for whole blood intracellular staining of IFN-γ, fixed with 4% paraformaldehyde and analyzed as above. Lymphocyte were firstly gated by distribution in flow cytometry distinguished from other cell community. For analyzed “CD27^−^CD4^+^” T cells, CD4^+^ T cells were gated and analyzed CD27 expression while “IFN-γ^+^CD4^+^” cells were gated in detecting CD27 of “CD27^−^IFN-γ^+^CD4^+^” cells. All flow cytometry results were analyzed by the Kaluza analysis software (USA) supported by Beckman Coulter company.

### Statistical analysis

All data analyses were performed using GraphPad Prism version 5.0 software (San Diego, CA). The difference between unpaired and paired samples was analysed using one-way ANOVA, *t*-test or chi-squared test. For the basic statistics for the patients enrolled in this study, percentiles and the mean ± SD were used. The sensitivity, specificity, AUC (area under curve) and 95% CI (95% confidence interval) were assessed by receiver operating characteristic (ROC) curve analysis. The association between 2 quantitative variables was measured using bivariate correlation (Spearman). A *P*-value of 0.05 was considered significant.

## Supplementary Information

**Additional file 1: Supplemental Figure 1**. The flow diagram. (a) The patient flow diagram. (b) The control flow diagram.

**Additional file 2: Supplemental Figure 2**. Relationship between the percentage of “CD27^−^IFN-γ^+^CD4^+^” expression and other clinical laboratory index. (a) Relationship between the percentage of “CD27^−^IFN-γ^+^CD4^+^” expression and ESR. (b) Relationship between the percentage of “CD27^−^IFN-γ^+^CD4^+^” expression and CRP. (c) Relationship between the percentage of “CD27^−^IFN-γ^+^CD4^+^” expression and IgA. (d) Relationship between the percentage of “CD27^−^IFN-γ^+^CD4^+^” expression and IgG. (e) Relationship between the percentage of “CD27^−^IFN-γ^+^CD4^+^” expression and IgM.

## Data Availability

The datasets used and /or analysed during the current study available from the corresponding author on reasonable request.

## Abbreviations

BMI: Body Mass Index
TSPOT.TB: Tuberculosis infectious T-lymphocyte spot assay
WBC: White Blood Cell counts
RBC: Red Blood Cell counts
HGB: Hemoglobin
PLT: Platelet counts
IgA: Immunoglobulin A
IgG: Immunoglobulin G
IgM: Immunoglobulin M
IgE: Immunoglobulin E
ESR: erythorcyte sedimentation rate
CRP: C-reaction protein
