# Supplementary figures and images for: Assessment of CD27 expression on T-cells as a diagnostic and therapeutic tool for patients with smear-negative pulmonary tuberculosis
Source: BMC Immunol. 2021 Jun 27;22:41. doi: 10.1186/s12865-021-00430-y (PMC8237462; doi:10.1186/s12865-021-00430-y)

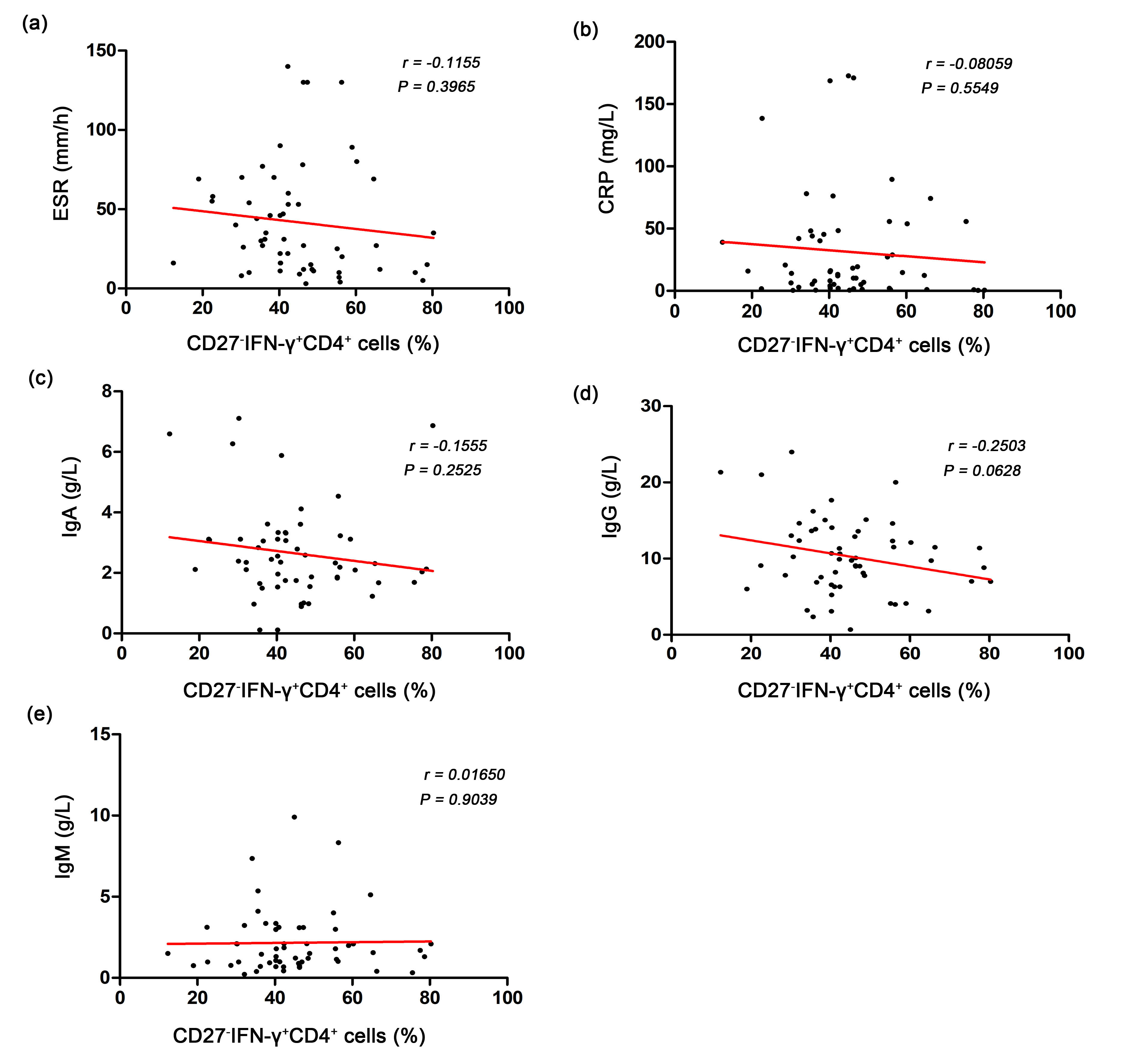

Supplement: Supplementary file 2 — Additional file 2: Supplemental Figure 2. Relationship between the percentage of “CD27−IFN-γ+CD4+” expression and other clinical laboratory index. (a) Relationship between the percentage of “CD27−IFN-γ+CD4+” expression and ESR. (b) Relationship between the percentage of “CD27−IFN-γ+CD4+” expression and CRP. (c) Relationship between the percentage of “CD27−IFN-γ+CD4+” expression and IgA. (d) Relationship between the percentage of “CD27−IFN-γ+CD4+” expression and IgG. (e) Relationship between the percentage of “CD27−IFN-γ+CD4+” expression and IgM. [file 12865_2021_430_MOESM2_ESM.tif]
